# A Comparison of PET Tracers in Recurrent High-Grade Gliomas: A Systematic Review

**DOI:** 10.3390/ijms24010408

**Published:** 2022-12-27

**Authors:** Sankar Muthukumar, Jordan Darden, James Crowley, Mark Witcher, Jackson Kiser

**Affiliations:** 1 Virginia Tech Carilion School of Medicine, Roanoke, VA 24016, USA; 2 Carilion Clinic Neurosurgery, Roanoke, VA 24016, USA; 3 Carilion Clinic Radiology, Roanoke, VA 24016, USA

**Keywords:** contrast-enhanced magnetic resonance imaging (CE-MRI), PET tracers, high-grade glioma

## Abstract

Humans with high-grade gliomas have a poor prognosis, with a mean survival time of just 12–18 months for patients who undergo standard-of-care tumor resection and adjuvant therapy. Currently, surgery and chemoradiotherapy serve as standard treatments for this condition, yet these can be complicated by the tumor location, growth rate and recurrence. Currently, gadolinium-based, contrast-enhanced magnetic resonance imaging (CE-MRI) serves as the predominant imaging modality for recurrent high-grade gliomas, but it faces several drawbacks, including its inability to distinguish tumor recurrence from treatment-related changes and its failure to reveal the entirety of tumor burden (de novo or recurrent) due to limitations inherent to gadolinium contrast. As such, alternative imaging modalities that can address these limitations, including positron emission tomography (PET), are worth pursuing. To this end, the identification of PET-based markers for use in imaging of recurrent high-grade gliomas is paramount. This review will highlight several PET radiotracers that have been implemented in clinical practice and provide a comparison between them to assess the efficacy of these tracers.

## 1. Introduction

Gliomas are the most commonly occurring tumors in the central nervous system, accounting for about 80% of all malignant primary brain tumors [1]. These neoplasms, which originate in the glial cells of the brain, are associated with high malignancy, recurrence risk and an extremely high mortality rate, and place a massive burden on patients, families, and society [2]. Gliomas can be classified into Grade I to IV based on the level of malignancy, which is determined by histopathological criteria.

High-grade gliomas are tumors diagnosed as either Grade III or Grade IV, and represent the most aggressive and invasive form of gliomas. These patients have a mean survival time of just 4–6 weeks with symptomatic care, and just 12–18 months with maximal tumor resection and appropriate adjuvant therapy [3]. Currently, the standard of care for high-grade gliomas involves three components: maximal surgical resection, external beam radiation therapy and chemotherapy with alkylating agents such as temozolomide [4]. However, despite this comprehensive treatment regimen, tumor recurrence occurs in patients within a median time of 6.7 months [5]. As a result, serial imaging is necessary to monitor the status of their disease and guide subsequent treatment. 

Currently, T1-weighted contrast-enhanced magnetic resonance imaging (CE-MRI) is the gold standard for image-guided diagnoses and treatment planning of recurrent high-grade gliomas [6]. In this imaging technique, images are obtained after an intravenous (IV) injection of a gadolinium-based contrast agent, with areas of brightness indicating sites of blood–brain barrier (BBB) disruption and vascular leakage caused by the tumor. The volume of the tumor as defined by CE-MRI serves as the basis for defining tumor burden and guiding treatment planning. Furthermore, CE-MRI has a significant role in evaluating treatment response after standard adjuvant therapy through the interpretation of changes in CE-MRI volumes with serial imaging. 

Despite its prevalent role in imaging of recurrent high-grade gliomas, CE-MRI has intrinsic limitations that may complicate the treatment of this condition. CE-MRI has been shown to underestimate tumor burden, as significant portions of tumors that do not disrupt the BBB lack contrast enhancement and, thus, cannot be visualized on imaging [6]. Failure to accurately delineate tumor volumes can result in undertreatment with surgical resection and adjuvant therapy, which contributes to poor clinical prognoses and recurrent tumors. Another major limitation CE-MRI presents is its inability to differentiate between tumor progression and treatment-related changes. While tumors are typically identified on CE-MRI as a result of BBB disruption, other processes which damage the BBB will present with the same contrast enhancement on CE-MRI, potentially complicating the diagnosis of tumor progression [7].

Treatment-related changes are often indistinguishable from tumor progression, displaying the similar enhancement patterns on CE-MRI. These changes involve pseudoprogression, an acute treatment response that manifests within 3–6 months, and radiation necrosis, a delayed response that is typically approximately 6–12 months after radiation therapy. Pseudoprogression has a reported incidence in 10–30% of patients undergoing radiation therapy, and is defined as radiographic evidence of disease progression with spontaneous resolution without further treatment [5,8]. It is believed to be due to factors causing tissue inflammation and the upregulation of vascular endothelial growth factor (VEGF), which lead to increased vessel permeability and edema. Patients exhibiting pseudoprogression show a favorable response to temozolomide chemotherapy treatment [9] and are symptomatically managed along with follow-up CE-MRI scans. 

Radiation necrosis is a delayed treatment effect caused by radiation damage to vascular endothelial cells and oligodendrocytes, leading to necrosis, reactive gliosis, and axonal swelling [10]. This condition is found in 3–24% of glioma patients who have received radiation therapy [11]. Compared to pseudoprogression, patients with radiation necrosis often experience more severe symptoms resulting in irreversible injury and significant neurological decline; thus, patients with radiation necrosis have a worse prognosis [5]. Patients with this condition often require medical or surgical intervention. 

In comparison to glioma patients exhibiting treatment-related changes on imaging, those with tumor progression experience a vastly different treatment course often requiring repeat surgical resection and alternative therapies, such as immunotherapy and tumor-treating fields (TTFields) [9]. As a result, making the distinction between these two courses is of paramount importance; failure to diagnose tumor progression may further worsen the prognosis of patients with high-grade gliomas and prevent them from receiving proper therapy, whereas the inability to identify treatment-related changes may lead to unnecessary surgery and therapy for tumor progression. Tumor recurrence will eventually begin to differentiate itself from treatment effect on imaging, but this may be at a point in time in which it may be too late to initiate effective treatment [12]. A recent meta-analysis showed that CE-MRI exhibited low sensitivity and specificity in detecting recurrent high-grade gliomas (68% and 77%, respectively) [13], suggesting that other imaging modalities to detect these tumors should be explored. 

### PET Overview

One of the primary alternatives to CE-MRI is positron emission tomography (PET), a diagnostic method that uses radiotracers to measure metabolic processes in the body. PET is particularly useful in oncology since cancer cells exhibit higher levels of metabolic activity than normal cells, and, thus, will display increased levels of radiotracer uptake on PET scans. Furthermore, this imaging modality can be combined with CE-MRI and CT to provide anatomical correlates for the metabolic information obtained from PET imaging. PET has been increasingly used in the evaluation of high-grade gliomas as it has demonstrated extensive utility in tumor grading, delineation of tumor extent, treatment planning and post-treatment assessment [14]. Due to its success in treatment planning, PET has potential use in guiding novel therapies such as intensity-modulated radiation therapy (IMRT) and laser interstitial thermal therapy (LITT). 

Notably, one of the key diagnostic properties of PET is its ability to differentiate tumor recurrence from treatment-related changes, which is one of the major limitations of CE-MRI. While CE-MRI requires the disruption of the BBB to localize tumors, PET radiotracers can be taken up through an intact BBB and can provide a greater approximation of tumor volume than CE-MRI. Given the poor prognosis of patients with high-grade gliomas and the high diagnostic benefits PET can provide, an exploration of different PET radiotracers is worth exploring. This review will provide a comparison of various tracers involved in the detection of recurrent high-grade gliomas and, if applicable, assess whether they provide better diagnostic information compared to the current gold standard of CE-MRI.

## 2. Results and Discussion

**Figure 1:**
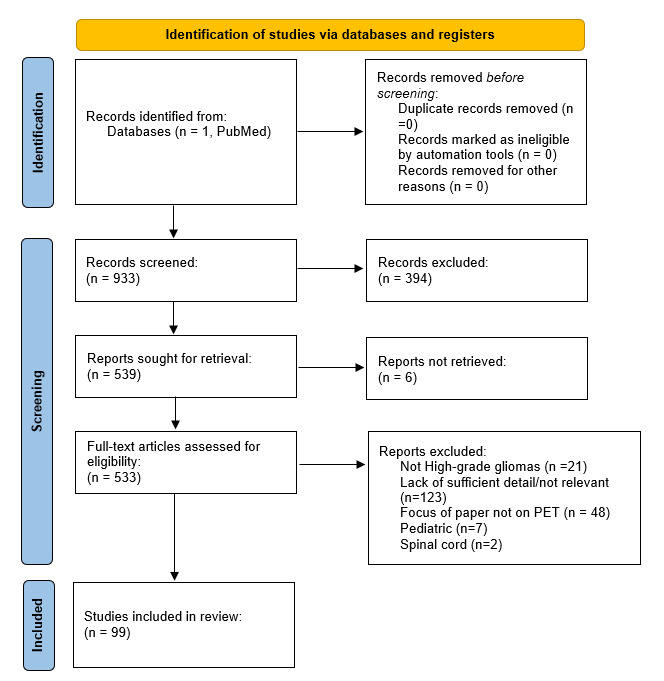
PRISMA study selection flowchart.

The PubMed search conducted for this review returned 933 papers (Figure 1). Of these, 394 were excluded after the initial screen, on the basis of the title and abstract. A secondary screen, which assessed full-text articles based on eligibility, yielded 332 papers. Studies were excluded based on whether they did not focus on high-grade gliomas (*n* = 21), did not provide sufficient detail (*n* = 123), did not discuss PET imaging (*n* = 48), involved pediatric cases (*n* = 7), and involved gliomas of the spinal cord (*n* = 2). After further screening, a final review yielded 99 papers. 

### 2.1. PET Radiotracers Used to Detect High-Grade Gliomas

#### ^18^F-fluorodeoxyglucose (^18^F-FDG)

PET scans traditionally utilize ^18^F-fluorodeoxyglucose (^18^F-FDG, a radiolabeled glucose analog) as a tracer to evaluate high metabolic activity [15]. This radiotracer has been used extensively to noninvasively assess the presence of many types of cancer, including those of the lung and breast, due to its long half-life (109.8 min) and wide availability. In the case of high-grade gliomas, ^18^F-FDG has demonstrated prognostic value, with its uptake levels serving as a strong predictor of survival time in patients with recurrent high-grade gliomas [16,17]. ^18^F-FDG uptake values have also been used to predict survival in high-grade glioma patients following antiangiogenic therapy using bevacizumab, suggesting a role in effectively evaluating treatment response [18]. 

Despite its extensive use in tumor diagnosis, ^18^F-FDG PET faces several limitations as an imaging modality. Most notably, high metabolic activity in normal cortical and deep gray matter causes increased ^18^F-FDG uptake in normal brain tissue, leading to a decreased tumor-to-background ratio in the detection of high-grade. This decreased contrast ratio makes it more difficult to detect tumors, particularly of high-grade glioma lesions of small size (<20 mm in diameter) [19]. Further complicating glioma diagnosis with ^18^F-FDG PET is the fact that glucose metabolism can be elevated at sites of inflammation, such as those caused by radiation, posing additional challenges for the diagnosis of tumor progression versus treatment-related changes. 

### 2.2. Amino Acid Tracers

Due to some of the drawbacks of ^18^F-FDG PET, studies have been conducted in recent years to assess novel PET radiotracers in the realm of high-grade gliomas. In particular, the use of amino acid PET tracers has emerged at the forefront of high-grade glioma imaging, primarily due to the high uptake of various amino acids in tumor tissue and low uptake in normal brain tissue [6]. These amino acid tracers are taken up through the L amino acid transport system (LAT1 and LAT2). LAT1 has increased expression in high-grade gliomas, making it a valuable target for PET imaging. Three of the most common substrates that pass through LAT1 are methionine, L-DOPA and L-tyrosine, all of which have an associated radiolabeled tracer in clinical practice for high-grade gliomas. The increased tumor-to-background ratio using amino acid PET imaging improves the discrimination of gliomas compared to ^18^F-FDG, which yields a high background activity. In the context of recurrent gliomas, amino acid radiotracers have demonstrated success in differentiating tumor progression from treatment-related changes, exhibiting higher sensitivity and specificity compared with ^18^F-FDG PET [20,21]. These tracers have been shown to be useful in the imaging of gliomas and other CNS malignancies.

#### 2.2.1. ^11^C-MET

The most well-established amino acid tracer in clinical practice is ^11^C-methionine (^11^C-MET), a tracer that is taken up into glioma tissue through LAT1. As with most amino acid tracers, ^11^C-MET exhibits a high detection rate of gliomas due to its low physiological uptake in normal brains, leading to an increased tumor-to-brain contrast ratio on imaging. However, with a half-life of just 20 min, the usage of this tracer is limited to locations with an on-site cyclotron. Another complicating factor in the diagnosis of recurrent high-grade gliomas is the increased uptake of methionine in radiation necrosis; it is believed that gliosis mediated by astrocytes and microglia leads to increased methionine metabolism [22]. 

^11^C-MET provides a modest level of diagnostic information for recurrent high-grade gliomas, with a recent meta-analysis showing a pooled sensitivity and specificity of 70% and 93% [23]. Another study found a sensitivity, specificity, and diagnostic accuracy in assessing tumor recurrence of 94.7%, 80%, and 89.6%, respectively, of high-grade gliomas [24]. In comparison to CE-MRI, ^11^C-MET demonstrated a higher sensitivity, while CE-MRI exhibited a higher specificity, although there was no statistically significant difference between either modality. 

Another major use of ^11^C-MET is in the evaluation of the treatment effect in GBM. Hirono et al. found that ^11^C-MET can be used to predict tumor progression in long-term GBM patients undergoing temozolomide therapy, as high uptake values after treatment correlated with a high recurrence rate [25]. This PET tracer has also been used to evaluate antiangiogenic therapy in glioblastoma (GBM) patients; ^11^C-MET uptake in patients undergoing bevacizumab treatment was also demonstrated to be a prognosticator of treatment success [26]. Given that bevacizumab has been shown to reverse the vascular permeability effects of BBB disruption, CE-MRI often fails at detecting tumor infiltration. Therefore, ^11^C-MET provides valuable treatment information in evaluating antiangiogenic therapy, which CE-MRI cannot do. 

^11^C-MET also has utility in evaluating glioma patients who undergo other forms of treatment. A study found that ^11^C-MET was useful in monitoring the short-term therapeutic effects of laser-induced interstitial thermotherapy (LITT) in the management of GBM patients, as a continuous decline in metabolic tumor volume indicated by ^11^C-MET correlated with treatment [27]. Regarding treatment planning, this radiotracer has been used to delineate tumor extent for re-irradiation, showing uptake outside the CE-MRI enhancement region in 74% of patients [28]. 

#### 2.2.2. ^18^F-FET

Another well-established radiotracer in clinical practice is O-(2-[18F]-fluoroethyl)-L-tyrosine (^18^F-FET), an L-tyrosine analog. Like ^11^C-MET, ^18^F-FET uptake takes place through LAT1. Some of the major advantages of ^18^F-FET include its long half-life (109.8 min), allowing it to be used at locations without an on-site cyclotron, and its ability to be taken up through an intact BBB. 

^18^F-FET has demonstrated effective diagnostic capabilities in differentiating high-grade glioma progression from pseudoprogression. A recent study of 22 patients with GBM showed a sensitivity of 100%, specificity of 91% and diagnostic accuracy of 96% in differentiating pseudoprogression from early tumor progression (within 12 weeks of radiotherapy), providing valuable information for the early detection of high-grade gliomas [29]. For these patients, TBR_max_ of ^18^F FET served as an effective prognosticator of overall survival, as a threshold of TBR_max_ < 2.3 predicted significantly longer overall survival time (23 months vs. 12 months). In a similar study, researchers used a tumor/white matter (T/Wm) cutoff of 2.5 for image interpretation in 72 patients, and a sensitivity of 80% and specificity of 87.5% were obtained [30]. In addition to discriminating early tumor progression from pseudoprogression, Kebir et al. showed a sensitivity of 84% and specificity of 86% of ^18^F-FET in differentiating late progression from treatment-related changes in patients with high-grade gliomas [31]. Finally, ^18^F-FET also has a role in evaluating treatment progression, as Piroth et al. found that early responders to radiochemotherapy (those whose TBR_max_ of ^18^F-FET uptake decreased by more than 10%) had a significantly longer median disease-free survival (10.3 vs. 5.8 months) than non-responders [32]. 

#### 2.2.3. ^18^F-FDOPA

Another amino acid tracer used in the clinical setting is 3,4-dihydroxy-6-(^18^F) fluoro-L-dihydroxy-phenylalanine (^18^F-FDOPA), which is also taken up through LAT1, and, thus, does not require disruption of the BBB for imaging. Originally used to evaluate presynaptic function in patients with neurodegenerative and movement disorders by targeting dopamine receptors in the brain, ^18^F-FDOPA has recently started being used for the diagnosis of gliomas. As mentioned previously, one of the major disadvantages to using ^18^F-FDOPA is its overexpression in the striatum; thus, its clinical utility in diagnosing gliomas within that region of the brain is limited. 

The use of ^18^F-FDOPA in detecting recurrent high-grade glioma is well-established. Youland et al. found that the sensitivity and specificity of ^18^F-FDOPA in diagnosing tumor recurrence was 85% and 80%, respectively [33]. Another study involving 24 patients obtained a sensitivity and specificity of 100% and 100%, respectively, in the detection of high-grade glioma [34]. Based on these data, ^18^F-FDOPA exhibits a high diagnostic accuracy in evaluating recurrent high-grade gliomas. 

^18^F-FDOPA has also been shown to be an important prognostic evaluator of recurrent glioma patients. One study found that the tumor-to-normal-brain ratio obtained from ^18^F-FDOPA imaging was an independent predictor of overall survival in patients with suspected high-grade glioma [35]. However, these findings differ from those of Herrmann et al., who noted that ^18^F-FDOPA tumor uptake accurately predicted progression-free survival but not overall survival [36]. In terms of evaluating treatment response, Harris et al. found that an increased uptake volume after antiangiogenic therapy was associated with a shortened progression-free survival in glioma patients [37]. It was also found that responders to bevacizumab treatment as assessed by ^18^F-FDOPA survived 3.5 times longer than non-responders [38]. 

#### 2.2.4. ^11^C- and ^18^F-Choline

Abnormal choline metabolism has recently emerged as a major hallmark of oncogenesis and tumor progression. To keep up with the growing demands of cancer metabolism, there is an increased uptake of choline in tumor tissue for various functions, such as synthesis of phospholipids in cell membranes [39]. As such, choline uptake has been used as a major target for PET, and recent studies have started to implement ^11^C- and ^18^F-choline imaging of different forms of cancer, including those of the brain. Overall, choline has been shown to have extremely low accumulation in normal brain tissue, making it a strong target for PET imaging. 

A recent study assessing ^11^C-choline in recurrent high-grade gliomas obtained a sensitivity of 100%, specificity of 70% and diagnostic accuracy of 81.3% [40]. When a T/N ratio of 1.42 was used as the cutoff for diagnosis, a sensitivity of 100%, specificity of 90% and diagnostic accuracy of 93.8% were noted, indicating a strong diagnostic performance. 

Like other amino acid tracers, ^18^F-choline PET can also be used to predict the survival of patients with high-grade glioma. Patients with a lower T/N ratio were found to have longer overall survival and progression-free survival times than those with a higher ratio [40]. Another study indicated that ^11^C-choline can be used to predict early treatment response in high-grade glioma patients treated with radiation therapy and temozolomide [41]. 

#### 2.2.5. ^18^F-FACBC

Anti-1-amino-3-18F-fuorocyclobutane-1-carboxylic acid (^18^F-FACBC), otherwise known as ^18^F-fluciclovine, is a recently developed synthetic amino acid radiotracer. As with other amino acid radiotracers, ^18^F-fluciclovine is transported into glial cells through LAT1 transporters but is also additionally taken up through alanine-cystine-serine transporters (ACSTs), which are also upregulated in cancer cells [42]. This radiotracer has traditionally been used for prostate cancer, but has emerged as a target for glioma imaging, particularly because it exhibits a high degree of accumulation in glioma tissue and a low degree in normal brain tissue. It has been shown to effectively discriminate between low and high-grade gliomas, with a sensitivity of 90.9% and a specificity of 97.5%; however, there have not been many studies evaluating this tracer in diagnosing tumor recurrence [43]. 

While there has not been a direct assessment of the diagnostic accuracy of ^18^F-fluciclovine for recurrent high-grade gliomas, recent studies have indicated that the tracer exhibits several properties, which would make it a promising target for glioma imaging. Compared to other PET amino acid tracers, including ^11^C-MET and ^18^F-FET, ^18^F-fluciclovine had an increased SUV_max_ (8.3) and T/N ratio (21.5), primarily due to its low accumulation in the healthy brain [44]. Due to this high contrast level of ^18^F-fluciclovine, even a very small lesion, such as a satellite tumor, may be detected with PET imaging, which would not be feasible with the use of CE-MRI [45]. Early detection of these small lesions is advantageous, as it allows for the early treatment of the tumor before it progresses. 

#### 2.2.6. ^11^C-AMT

α-11C-methyl-L-tryptophan (^11^C-AMT) is a radiotracer that was originally developed to assess the synthesis of serotonin in the brain using a precursor amino acid, tryptophan, in the evaluation of various psychiatric conditions. Recently, this tracer has also been used for cancer imaging, as the conversion of tryptophan into kynurenine metabolites is upregulated, and, thus, tryptophan uptake by tumor tissue is increased in oncogenic conditions [46]. As such, studies have shown increased uptake of ^11^C-AMT in high-grade gliomas, suggesting it can be used as a diagnostic biomarker in this condition [47]. 

As with ^18^F-fluciclovine, there have not been studies conducted to evaluate the performance of ^11^C-AMT in diagnosing glioma recurrence. However, in preliminary studies evaluating this tracer, it was found that changes in SUV_max_ after treatment were predictive of overall survival in high-grade gliomas [48]. Furthermore, one study that used ^11^C-AMT in conjunction with CE-MRI found that PET volumes more accurately delineated recurrent tumor extent compared to CE-MRI, suggesting a role in subsequent treatment planning [49]. Similarly, gross tumor volume as defined by ^11^C-AMT-PET was shown to produce superior recurrence volume compared to that defined by CE-MRI [50]. 

#### 2.2.7. ^18^F-FBY

^18^F-Tyr-BF3 (^18^F-FBY) is another LAT1-targeting PET tracer, which has recently been introduced to glioma imaging. This tracer has been found to have decreased activity in normal brain compared to other LAT1-based tracers, such as ^18^F-FET, leading to increased contrast on imaging high-grade gliomas (TBR_mean_ of 24.56) [51]. This increased contrast ratio may help with the early detection of recurrent high-grade gliomas and guide the subsequent treatment for patients, though it will require further investigation and validation of this tracer. 

#### 2.2.8. ^18^F-FBPA

Amino acid tracer 4-borono-2-[^18^F]fluorophenylalanine (^18^F-FBPA) has been recently introduced and has a high selectivity for the LAT1 transport system, and, thus, exhibits higher uptake in cancerous tissue. Beshr et al. compared uptake levels of recurrent high-grade gliomas with those of radiation necrosis and found a significantly higher increase in SUV_max_ of high-grade gliomas compared to necrosis (4.63 to 1.93) [52]. As such, ^18^F-FBPA may serve as a promising tracer for the detection of recurrence once it has been validated and explored further.

#### 2.2.9. ^18^F-FGln

Another novel radiotracer that has been studied in glioma patients is 18F-(2S,4R)-fluoroglutamine (^18^F-FGln). Glutamine is the most abundant amino acid in plasma, and has an essential role in cell growth, due to its role in synthesizing proteins, lipids and nucleic acids. Due to increased metabolic needs, glutamine uptake is increased in cancer cells, contributing to tumor growth and proliferation. As a result of this increased uptake, glutamine-based radiotracers, such as ^18^F-FGln, may have a valuable role in imaging high-grade gliomas. In an initial investigation of ^18^F-FGln PET imaging by Venneti et al., increased 18F-FGln uptake was noted in the three subjects with gliomas, along with minimal uptake in normal brain tissue, with TBR values ranging from 3.7–4.8 [53]. These values suggest a potential role in the imaging of high-grade gliomas by ^18^F-FGln after it has been investigated further as a diagnostic tool. 

### 2.3. Hypoxia Tracers

Another major characteristic of tumor growth is the presence of hypoxia in the local tumor environment; tumor cells respond to hypoxia by acquiring a more metastatic and aggressive phenotype. Consequently, PET radiotracers that target areas of hypoxia have been developed and implemented clinically for the diagnosis of tumors such as gliomas. One of the major advantages of using hypoxia tracers is that it can provide an early assessment of tumor progression after treatment as hypoxic changes take place relatively quickly [54]. 

### 2.3.1. ^18^F-FMISO

^18^F-fluoromisonidazole (^18^F-FMISO) is the most widely studied hypoxia tracer in PET imaging. Due to its lipophilicity, it passively diffuses through the cell membrane and is reduced into R-NO_2_ radicals once inside. In a well-oxygenated environment, the tracer can diffuse back into the extracellular space, but in hypoxic conditions, the reduction process continues, leading to ^18^F-FMISO buildup in the cell [55]. As the degree of hypoxia is intrinsically tied to increased tumor progression and aggressiveness, ^18^F-FMISO appears to be a promising target for the diagnosis of high-grade gliomas. A recent study found that ^18^F-FMISO provided a better estimation of tumor extent in GBM than CE-MRI, suggesting a role in treatment planning [56]. Furthermore, hypoxia in the tumor microenvironment leads to stimulation such as VEGF, promoting angiogenesis. Barajas et al. found that recurrent high-grade glioma patients who underwent bevacizumab therapy showed a marked decrease in ^18^F-FMISO uptake, validating this potential role signifying a potential role of ^18^F-FMISO in monitoring tumor progression during antiangiogenic therapy [57]. 

### 2.3.2. ^18^F-FETNIM

Another hypoxia used in glioma imaging is ^18^F-fluoroerythronitroimidazole (^18^F-FETNIM), which, like ^18^F-FMISO, is a derivative of nitroimidazole. Compared to ^18^F-FMISO, this tracer is more hydrophilic, resulting in lower background signals and, therefore, an increased tumor-to-brain contrast ratio on PET imaging [58]. Hu et al. demonstrated that ^18^F-FETNIM had a greater SUV_max_ in these high-grade gliomas compared to low-grade and normal brain tissue, which was correlated with the expression of various angiogenic growth factors [59]. Furthermore, another study revealed that the overall survival of patients was significantly shorter in patients with a higher T/B ratio than in those with lower uptake [60], indicating a prognostic role of ^18^F-FETNIM. 

### 2.3.3. ^18^F-FAZA

^18^F-flouroazomycin arabinoside (^18^F-FAZA) has also been investigated as a hypoxia tracer in PET glioma imaging. Like the two aforementioned hypoxia radiotracers, this tracer is part of the 2-nitroimidazole group and is taken up through passive diffusion due to its lipophilic properties. ^18^F-FAZA also exhibits increased imaging contrast compared to ^18^F-FMISO due to its low uptake in the healthy brain; however, it does not accumulate in tumor regions without BBB disruption [61]. Although clinical studies examining ^18^F-FAZA have been limited, this tracer can identify tumor areas with the highest grade, potentially providing a more effective target for therapy [62]. 

### 2.3.4. ^64^Cu-Cu(ATSM)

One unconventional tracer used to measure hypoxia is Cu-diacetyl-bis (N4-methylthiosemicarbazone, otherwise known as [^64^Cu][Cu(ATSM)]. The mechanism of uptake of this tracer is still not well-understood, but it is thought that after passive diffusion through the cell membrane, Cu(II)-ATSM is typically reduced to Cu(I)-ATSM, but with hypoxic conditions, Cu(I)-ATSM is subsequently dissociated into H_2_-ATSM and free Cu(I) [63]. The radiolabeled copper is evaluated on imaging, and, therefore, is highly expressed in areas of poor oxygenation, such as cancer cells. Compared to the nitroimidazole-based tracers, [^64^Cu][Cu(ATSM)] it shows higher uptake, generating high-quality images as soon as 20 min after injection [64]. Regarding imaging of high-grade gliomas, it was found that tumor uptake was correlated with hypoxia-inducible factor 1α, a biomarker of poor oxygenation. Furthermore, the same study found that higher uptake of this tracer in high-grade glioma patients correlated with poor treatment response and poor diagnosis, suggesting a role for this tracer in glioma treatment planning. 

### 2.4. Other Tracers: Proliferation Markers

#### ^18^F-FLT

^18^F-fluoro-3′-deoxy-3′-L-fluorothymidine, otherwise known as ^18^F-FLT, has been developed to measure thymidine kinase-1 activity, which is a marker for tumor proliferation rate as it is one of the major enzymes of DNA synthesis. Unlike the amino acid tracers, this tracer does not readily cross the BBB, and, thus, its uptake is dependent on BBB disruption [65]. As a result, the distribution of this tracer is very similar to the region of contrast-enhancement presented on CE-MRI. 

One complicating factor with ^18^F-FLT is that since it requires BBB disruption, other factors that increase the vascular permeability of the BBB, such as radiotherapy, may contribute to the degree of ^18^F-FLT uptake [66]. In addition to prognostic value, ^18^F-FLT may, potentially, have a role in monitoring treatment progression. A preclinical study involving temozolomide, a chemotherapy drug, found that ^18^F-FLT may be effective in evaluating treatment response for GBM [67], although more investigation is required to assess clinical efficacy.

### 2.5. Anti-Apoptotic Markers

#### ^18^F-GE-180

^18^F-GE-180 is a PET tracer that targets the translocator protein (TSPO) found in the outer mitochondrial membrane of cells, namely, activated astrocytes and microglia. This protein has a complex role in tumor metabolism, which is not fully understood, yet it is highly expressed in tumors such as high-grade gliomas [68]. Notably, these tumors have barely any expression in the healthy brain, leading to a higher tumor-to-brain ratio in gliomas [69], which is important for early diagnosis and treatment. ^18^F-GE-180 has also been shown to delineate tumor volume beyond that of CE-MRI, making it a promising target for further study [70]. 

### 2.6. Other Tracers

#### 2.6.1. PSMA-Based Radiotracers

Prostate-specific membrane antigen (PSMA), also known as glutamate carboxypeptidase II, is another valuable target for cancer imaging due to its overexpression in prostate cancer. Due to the overexpression of PSMA in prostate cancer cells, this tracer has traditionally been used to image that condition. However, recent observations have shown that PSMA is also overexpressed in high-grade glioma neovasculature, indicating that PSMA PET radiotracers can serve as an effective diagnostic tool in this realm as well [71]. ^18^F-DCFPyL is a PSMA tracer that binds PSMA in both GBM and anaplastic astrocytoma, making it a valuable imaging target [72]. Additionally, ^18^F-PSMA-1007 has demonstrated significant uptake in recurrent high-grade gliomas, and, thus, may have some clinical utility [73]. While these and other PSMA tracers have been used to image gliomas in some capacity, ^68^Ga-PSMA-11 is the most extensively studied in the domain of high-grade gliomas. Due to its extremely low uptake in normal brain tissue, ^68^Ga-PSMA-11 has a very high tumor-to-brain ratio in high-grade gliomas (median value of 96.7), surpassing that of amino acid tracers such as ^18^F-FET and ^18^F-FDOPA [74]. This high contrast ratio has been suggested to be advantageous in distinguishing treatment-related changes from tumor progression in GBM patients. Finally, ^68^Ga-PSMA-11 may have a particular use in diagnosing early tumor recurrence as it has been shown to be poorly expressed in treatment-related changes such as radiation necrosis [75]. 

##### 2.6.2. ^13^N-Ammonia

^13^N-Ammonia is a radiotracer that measures levels of regional blood flow in tissues. Since tumors are highly metabolic, they exhibit increased perfusion, and, therefore, ^13^N-Ammonia uptake is increased in cancer cells. Traditionally, this tracer was used to image perfusion in the myocardium of the heart but has recently been introduced in glioma imaging, possibly due to the involvement of ammonia in glutamine synthesis [76]. It has been suggested that since treatment-related changes are not associated with increased metabolism, ^13^N-Ammonia may be an effective tool in discriminating between tumor and treatment effects. 

#### 2.6.3. FAPI Radiotracers

Fibroblast activation protein (FAP) is a serine peptidase that is overexpressed in cancer-associated fibroblasts, and radiotracers that bind to these proteins (such as FAP inhibitors) may serve as an effective target in imaging high-grade gliomas [77]. A recent study by Huang et al. showed that high-grade gliomas show high uptake of FAPI and low uptake in normal brain tissue, indicating potential use for this tracer in diagnosing high-grade gliomas [78]. In addition to detecting gliomas, FAPI imaging agents also showed high TBR for brain metastases from other cancers, including those from the liver, stomach, breast, and lung. 

#### 2.6.4. Miscellaneous Tracers

^124^I-CLR1404 is a recently developed alkyl phosphocholine analog that enters cells primarily through lipid rafts in plasma membranes. These lipid rafts are overexpressed six- to ten-fold in malignant cells compared to normal cells, making this tracer an ideal target for cancer imaging, particularly with gliomas [79]. ^124^I-CLR1404 exhibits a high TBR of 10.04, which compares favorably with other PET tracers in glioma research. However, in comparison with CE-MRI imaging, this tracer showed both concordant and discordant areas of ^124^I uptake, suggesting that further investigation is needed to validate the use of this tracer in high-grade gliomas. 

^18^F-FPPRGD2 is a radiotracer associated with the integrin α_v_β_3_, which is overexpressed in several types of cancer cells, including gliomas [80]. Initially tested in breast cancer, this tracer was recently utilized for the first time in GBM PET imaging [81]. This study found that ^18^F-FPPRGD2 may serve as a moderately good evaluator of treatment response, as GBM patients exhibiting an early decrease in SUV_max_ of this tracer after bevacizumab therapy had a better prognosis than non-responders. However, as there are limited findings involving this radiotracer in high-grade glioma, more studies are required to establish its diagnostic efficacy. 

Tracer 68Ga-BZH3 binds to neuromedin B (BB_1_), a bombesin receptor that is overexpressed in several GBM cell lines [82]. As with ^18^F-FPPRGD2, not many studies have been conducted to evaluate this tracer in glioma imaging, so more evidence is needed to establish the effectiveness of this tracer. That said, one study indicated that ^68^Ga-BZH3 is overexpressed in recurrent high-grade gliomas and can be used to differentiate high-grade from low-grade gliomas, suggesting that its role in diagnosis may be worth exploring [83]. 

## 3. Discussion

To address the limitations of CE-MRI in diagnosing recurrent high-grade gliomas, PET imaging has been recommended as an alternative imaging modality. To this end, certain well-established radiotracers have consistently demonstrated high diagnostic accuracy in conjunction with and separate from CE-MRI imaging for glioma patients, making it easier to detect the presence of the tumor and distinguish it from treatment-related changes so that patients receive treatment earlier and improve their prognosis. As these tracers become more widespread in clinical practice, assessing their efficacy in comparison to the gold standard of CE-MRI and with each other is necessary to determine their use in clinical practice (Table 1). 

### 3.1. Comparison with CE-MRI

Compared to the gold standard of CE-MRI, ^18^F-FDG has been shown to provide modest diagnostic utility. One study of 90 patients with suspected recurrent gliomas showed that ^18^F-FDG PET demonstrated higher specificity in the detection of recurrence in high-grade gliomas than CE-MRI (100% to 25% in grade IV and 83.3% to 33% in grade III, respectively) [84]. A recent meta-analysis of recurrence detection in high-grade gliomas by ^18^F-FDG indicated a summary sensitivity of 79% and summary specificity of 70% [23]. These values correspond to a positive likelihood ratio of 2.6 and negative likelihood ratio of 0.3, indicating a moderate diagnostic ability. As mentioned, a major limitation of ^18^F-FDG compared to other radiotracers in clinical practice is that the brain is normally a very metabolically active organ, and, thus, the difference in uptake levels between normal brain tissue and ^18^F-FDG is unsubstantial.

However, when used in conjunction with various CE-MRI imaging modalities, ^18^F-FDG PET provides additive diagnostic value. In a recent study, it was shown that the prognostic accuracy in predicting survival in recurrent GBM patients increased from 58% with CE-MRI alone to 74% with a combination of ^18^F-FDG PET and CE-MRI [85]. Additionally, a comparison between different imaging modalities of recurrent GBM involving 24 patients found that ^18^F-FDG PET/MRI combined with dynamic susceptibility contrast (DSC) perfusion MRI provided the best diagnostic capabilities in distinguishing tumor- from treatment-related changes, yielding a sensitivity and specificity of 100% [86]. 

While ^18^F-FDG provides a limited degree of additional diagnostic information, amino acid tracers generally have demonstrated better success in comparative studies with CE-MRI. A posttreatment study found that ^18^F-FET was able to detect treatment failure to antiangiogenic therapy earlier than CE-MRI, indicating that this tracer may also have a role in guiding treatment for high-grade gliomas [87]. Early glioma detection is paramount, as it allows patients to initiate vital treatments earlier, which may prove to be lifesaving before the tumor burden grows. ^18^F-FET PET has also exhibited a higher sensitivity in detection of residual tissue after GBM resection compared with CE-MRI [88]. It has been shown that greater extent of tumor resection is associated with a longer overall survival time in glioma patients; therefore, the localization and resection of residual tumor may lead to better outcomes for these individuals [89]. As with ^18^F-FDG, the combined use of ^18^F-FET PET / MRI has been shown to be more effective than either one individually. Lundemann et al. demonstrated that the composite tumor volume assessed by CE-MRI and ^18^F-FET PET was a better predictor for the localization of GBM recurrence than either alone [90], allowing for more effective treatment planning and failure analysis. Additionally, one study found that when ^18^F-FET PET imaging was used in the management of patients with recurrent high-grade glioma treated with bevacizumab and irinotecan (two chemotherapy drugs), the rate of correct diagnoses of tumor progression compared to treatment-related changes increased by 41.4% [91]. 

The number of studies comparing other PET radiotracers with CE-MRI is limited, yet those that have been tested have shown modest improvement over CE-MRI in diagnosing high-grade gliomas. Youland et al. showed that ^18^F-FDOPA imaging was better at predicting tumor recurrence than CE-MRI, using a T/N ratio > 2.0 and SUV_max_ > 1.36. A comparison between ^68^Ga-PSMA-11 and CE-MRI showed higher sensitivity in diagnosing high-grade gliomas than CE-MRI (85% to 71%), although both had comparable specificities [92]. ^18^F-FLT was found to be slightly more effective at assessing early tumor progression than CE-MRI and was a strong predictor of progression-free and overall survival [93]. A study of 14 patients with recurrent high-grade glioma found that ^13^N-ammonia had a higher diagnostic accuracy than CE-MRI (78.6% to 71.4%) [94]. Lastly, a study involving 55 patients showed a sensitivity of 92% and specificity of 87.5% for ^11^C-choline imaging, which were higher than those of CE-MRI evaluated in the same study [95]. 

### 3.2. Comparison between Tracers 

While it is important to assess various tracers in comparison to CE-MRI, a comprehensive evaluation of which tracer is best in diagnosing recurrent high-grade gliomas is also warranted (Table 2). Generally, most of the studies involving recently developed tracers have provided a direct comparison with ^18^F-FDG and have demonstrated superior performance in diagnosing recurrent high-grade gliomas. The most used amino acid tracer, ^11^C-MET, was the first to demonstrate superior performance to ^18^F-FDG, with Takenaka et al. showing that ^11^C-MET has a higher accuracy than ^18^F-FDG in diagnosing glioma recurrence [32]. 

Other amino acid tracers have also compared favorably to ^18^F-FDG in diagnosing recurrent high-grade gliomas. A study involving 55 patients showed a sensitivity of 92% and specificity of 87.5% for ^11^C-choline imaging, which were higher than those of ^18^F-FDG evaluated in the same study [95]. In a comparison of ^18^F-FET to ^18^F-FDG, it was found that ^18^F-FET provided a better delineation of tumor extent and increased uptake in high-grade gliomas, potentially having more utility in treatment planning and clinical management [96]. As mentioned before, tracers using ^11^C, such as ^11^C-MET and ^11^C-CHO, have a very short half-life of just 20 min, limiting their use to institutions with an on-site cyclotron. As a result, although ^18^F-FET and ^11^C-MET exhibit similar diagnostic performance in diagnosing recurrent high-grade gliomas according to recent literature, the longer half-life of ^18^F-FET enables it to be used more broadly [97]. Lastly, ^18^F-FDOPA was shown by Chen et al. to have a higher diagnostic accuracy in the evaluation of recurrent tumors than ^18^F-FDG [98]. 

Non-amino acid tracers have also performed well in comparison to ^18^F-FDG in this realm. A study by Chen et al., which studied 25 patients with recurrent high-grade tumors, validated the prognostic ability of this radiotracer for patient survival, and found that ^18^F-FLT was more sensitive than ^18^F-FDG in imaging this condition [99]. The study also found that ^18^F-FLT correlated better with Ki-67 values, a marker of active cell proliferation. ^13^N-ammonia and ^68^Ga-PSMA-11 were also found to be superior to ^18^F-FDG in detecting recurrent astrocytoma, showing increased uptake values and increased contrast ratios [76,100]. 

Comparisons between non-FDG tracers have been performed in recent years and show definitive advantages of certain radiotracers over others. One study comparing ^18^F-FET to ^18^F-FLT, a marker of cellular proliferation, found that ^18^F-FET but not ^18^F-FLT could detect tumor tissue beyond that found on CE-MRI. Additionally, ^18^F-FLT uptake requires BBB disruption unlike ^18^F-FET, which can be taken up through an intact BBB [101]. A comparison between ^18^F-FET and ^18^F-FDOPA, another amino acid tracer, in recurrent high-grade gliomas revealed a significantly higher SUV_max_ for ^18^F-FET (4.9 vs. 4.3) for ^18^F-FDOPA; however, both performed equally well in delineating tumor progression [102]. However, ^18^F-FDOPA exhibits high uptake in the striatum, making the diagnosis of tumors in those areas complicated with this radiotracer. Despite this limitation, ^18^F-FDOPA was found to be superior to ^13^N-Ammonia in detecting recurrence [103,104]. ^18^F-FDOPA also provided a similar level of diagnostic accuracy as ^11^C-MET, although the use of ^18^F over ^11^C provides numerous advantages in clinical practice.

Based on these findings, ^18^F-FET appears to provide the most advantages of any PET tracer, and, thus, should be the primary radiotracer used to detect recurrent high-grade gliomas at this point in time. As mentioned above, ^18^F-FET is one of the more well-validated tracers in clinical practice, has a long half-life, and can be taken up through an intact BBB. This PET tracer performs well in comparison with CE-MRI, showing a superior diagnostic performance and demonstrating utility in treatment planning and evaluation. Furthermore, ^18^F-FET has demonstrated a superior diagnostic performance than other commonly used tracers, such as ^18^F-FDG, ^18^F-FLT and ^18^F-FDOPA. Finally, ^18^F-FET imaging allows for the early differentiation of tumor presence from treatment-related changes, leading to earlier treatment and improved patient prognosis. While more comparative studies need to be conducted between various PET radiotracers, the current data suggest that ^18^F-FET may be the most effective in the clinical setting. 

### 3.3. Limitations

Despite the general success of these tracers in diagnosing recurrent high-grade gliomas, the number of studies assessing these radiotracers is still limited. Without significant data providing a direct comparison between them and CE-MRI, it is more difficult to assess their efficacy. Furthermore, while it is apparent that a combination of PET and CE-MRI generally provides the strongest diagnostic efficacy compared to either modality individually, the number of institutions with PET/CE-MRI scanners is fairly small, so the amount of data involving PET/CE-MRI is limited.

## 4. Methods

A literature search was conducted on the PubMed database to identify research studies involving the use of PET radiotracers in the detection of recurrent high-grade gliomas. The literature review was performed in accordance with the Preferred Reporting Items for Systematic Reviews and Meta-Analyses (PRISMA) guidelines, which were used to identify papers and screen them for inclusion or exclusion. The PubMed search was performed in August 2022 using the following algorithm: ((positron emission tomography) OR (PET) OR (PET/CT) OR (PET/MRI)) AND (((glioblastom*) OR (GBM)) OR (((high-grade) OR (grade III) OR (grade IV) OR (high grade) OR (anaplastic)) AND ((brain tumor) OR (CNS tumor) OR (neurooncology) OR (neuro-oncology) OR (gliom*) OR (astrocytom*) OR (oligodendrogliom*)))) AND ((recur*) or (radiation) or (necrosis) or (pseudoprogression)). All available dates were included in the search. 

## 5. Conclusions

In recent years, many imaging techniques have been employed in order to address the issue of diagnosing recurrent high-grade gliomas, given the dire prognosis of patients with this condition. Factors such as pseudoprogression and radiation necrosis appear to further complicate this diagnosis, appearing indistinguishable from tumor growth on CE-MRI, the standard imaging modality. Recent advancements in PET imaging have introduced new radiotracers into clinical practice, which have been able to address some of the limitations of CE-MRI, notably the ability to distinguish between tumor recurrence and treatment-related changes. As of now, amino acid tracers, such as ^11^C-MET, ^18^F-FET and ^18^F-FDOPA, have been the most studied and have been shown to successfully diagnose tumor recurrence early in a multitude of studies. Meanwhile, other tracers employing different mechanisms of action have also begun to demonstrate success in clinical practice. In the near future, a combination of PET and CE-MRI for diagnosis may show the most promise in accurate identification of recurrent high-grade gliomas, yet the success of PET as an independent imaging modality suggests that further evaluation is certainly worth pursuing.

## Figures and Tables

**Table 1:** Summary of PET tracers for recurrent high-grade glioma use.

Tracer	Mechanism of Action	Advantages	Drawbacks
18F-FDG	Glucose metabolism	Widely available	High uptake in normal brain tissue Limited diagnostic utility in high-grade gliomas
11C-MET	Amino acid transport	Treatment planning and evaluation Good diagnostic ability for recurrent high-grade gliomas	Short half-life, limited to use in on-site cyclotron
18F-FET	Amino acid transport	Long half-life Good diagnostic ability for recurrent high-grade gliomas Predictive of overall survival Treatment planning and evaluation	Limited availability
18F-FDOPA	Amino acid transport	Treatment planning and evaluation Good diagnostic ability for recurrent high-grade gliomas Predictive of progression-free survival	Striatal uptake Limited availability
18F-Choline	Amino acid transport	Good diagnostic ability for recurrent high-grade gliomas Treatment evaluation Long half-life	Limited availability
11C-Choline	Amino acid transport	Good diagnostic ability for recurrent high-grade gliomas Treatment evaluation	Short half-life Limited availability
18F-FACBC	Amino acid transport	Glioma grading Extremely high tumor-to-brain ratio	Not yet validated for diagnostic performance
11C-AMT	Amino acid transport	Predictive of overall survival	Not yet validated for diagnostic performance Short half-life, limited to use in on-site cyclotron
18F-FBY	Amino acid transport	Moderately high tumor-to-brain ratio	Not yet validated for diagnostic performance
18F-FBPA	Amino acid transport	Moderately high uptake values in tumor tissue	Not yet validated for diagnostic performance
18F-FGln	Amino acid transport	Moderately high uptake values in tumor tissue	Not yet validated for diagnostic performance
18F-FMISO	Hypoxia marker	Can identify small hypoxic tumor areas Treatment evaluation	Not yet validated for diagnostic performance
18F-FETNIM	Hypoxia marker	Can identify small hypoxic tumor areas Predictive of overall survival	Not yet validated for diagnostic performance
18F-FAZA	Hypoxia marker	Can identify small hypoxic tumor areas	Requires BBB disruption Not yet validated for diagnostic performance
64Cu-ATSM	Hypoxia marker	Can identify small hypoxic tumor areas Evaluates treatment response	Not yet validated for diagnostic performance
18F-GE-180	Neuroinflammation	Relatively high tumor-to-brain ratio	Not yet validated for diagnostic performance
18F-FLT	DNA synthesis	Treatment progression and prognosis	Requires damaged BBB
68Ga-PSMA-11	Glioma neovasculature	Extremely high tumor-to-brain ratio Treatment evaluation	Not yet validated for diagnostic performance in gliomas
13N-Ammonia	Tissue perfusion	Moderately high diagnostic accuracy	Short half-life
124I-CLR1404	Lipid raft transport	Moderately high tumor-to-brain ratio	Not yet validated for diagnostic performance
18F-FPPRGD2	Tumor angiogenesis	Treatment evaluation	Not yet validated for diagnostic performance
68Ga-BZH3	Tumor Angiogenesis	Glioma grading	Not yet validated for diagnostic performance
FAP	Fibroblast growth	Glioma grading	Not yet validated for diagnostic performance

**Table 2:** Summarized reported ranges of diagnostic information for recurrent high-grade gliomas using PET.

Tracer/Imaging Modality	Sensitivity	Specificity	Diagnostic Accuracy
CE-MRI	68%	77%	N/A
FDG	79%	70%	N/A
11C-MET	94.7%	80%	89.6%
18F-FET	80–100%	86–91%	96%
18F-FDOPA	85–100%	80–100%	N/A
11C-CHO	80–100%	70–100%	81.3–93.8%

## Data Availability

Not applicable.
